# Genes Associated with Altered Brain Structure and Function in Obstructive Sleep Apnea

**DOI:** 10.3390/biomedicines12010015

**Published:** 2023-12-20

**Authors:** Yijie Huang, Chong Shen, Wei Zhao, Youlan Shang, Yisong Wang, Hui-Ting Zhang, Ruoyun Ouyang, Jun Liu

**Affiliations:** 1 Department of Radiology, The Second Xiangya Hospital, Central South University, Changsha 410011, China; 218211072@csu.edu.cn (Y.H.); wei.zhao@csu.edu.cn (W.Z.); shangyoulan1028@163.com (Y.S.); wysdoctor@163.com (Y.W.); 2 Department of Respiratory and Critical Care Medicine, Second Xiangya Hospital, Central South University, Changsha 410011, China; 218202067@csu.edu.cn; 3 Clinical Research Center for Medical Imaging, Changsha 410011, China; 4 Department of Radiology Quality Control Center, Changsha 410011, China; 5 MR Research Collaboration Team, Siemens Healthineers, Wuhan 430000, China; huiting.zhang@siemens-healthineers.com

**Keywords:** resting-state fMRI, amplitude of low-frequency fluctuation, gray matter volume, transcriptomics, imaging biomarkers

## Abstract

Obstructive sleep apnea (OSA) has been widely reported to cause abnormalities in brain structure and function, but the genetic mechanisms behind these changes remain largely unexplored. Our research aims to investigate the relationship between sleep characteristics, cognitive impairments, genetic factors, and brain structure and function in OSA. Using structural and resting-state functional magnetic resonance imaging data, we compared cortical morphology and spontaneous brain activity between 28 patients with moderate-to-severe OSA and 34 healthy controls (HCs) utilizing voxel-based morphology (VBM) and the amplitude of low-frequency fluctuations (ALFF) analyses. In conjunction with the Allen Human Brain Atlas, we used transcriptome-neuroimaging spatial correlation analyses to investigate gene expression patterns associated with changes in gray matter volume (GMV) and ALFF in OSA. Compared to the HCs, the OSA group exhibited increased ALFF values in the left hippocampus (t = 5.294), amygdala (t = 4.176), caudate (t = 4.659), cerebellum (t = 5.896), and decreased ALFF values in the left precuneus (t = −4.776). VBM analysis revealed increased GMV in the right inferior parietal lobe (t = 5.158) in OSA. Additionally, functional enrichment analysis revealed that genes associated with both ALFF and GMV cross-sampling were enriched in gated channel activity and synaptic transmission, glutamatergic synapse, and neuron.

## 1. Introduction

Obstructive sleep apnea (OSA) is a prevalent sleep disorder [1] characterized by snoring, recurrent interruptions in sleep, fluctuations in oxygen levels, and excessive daytime drowsiness. The prevalence of OSA has experienced a significant increase in recent times [2], and it frequently coexists with numerous complications, giving rise to cardiovascular and cerebrovascular disorders, hypertension, as well as psychiatric ailments such as cognitive disorders associated with memory [3]. However, these aspects have not yet received sufficient attention. The pathophysiological mechanisms of OSA have yet to be fully elucidated, despite intermittent hypoxia being commonly regarded as an underlying process leading to oxidative stress and free radical production [4].

Neuroimaging techniques are increasingly employed as a noninvasive modality to elucidate the structural and functional disparities within the brain. In a previous study [5], resting-state functional magnetic resonance imaging (fMRI) was employed to quantify alterations in the amplitude of low-frequency fluctuation (ALFF) and regional homogeneity (ReHo), aiming to investigate changes in brain function among pediatric individuals with obstructive sleep apnea (OSA), while also exploring their correlation with neurocognitive dysfunction. Another investigation [6] employed two distinct yet complementary methodologies, namely cortical thickness analysis (CTA) and voxel-based morphometry (VBM), to evaluate disparities in gray matter thickness within the insula and cingulate regions between individuals with OSA and healthy controls. However, the majority of previous studies have predominantly focused on a singular mode of investigation. It is crucial to acknowledge that there exists a substantial level of synergy between brain structure and function, which collectively contribute to the development and progression of diseases and neurocognitive function [7]. Therefore, it is imperative to investigate the aberrations in brain structure and function among individuals with OSA and their correlation with neurocognitive performance using multimodal imaging techniques that integrate resting-state MRI, VBM, as well as specific alterations in ALFF and gray matter volume (GMV).

A recent study [8] conducted in China has identified novel and significant genetic loci associated with OSA and objective sleep-related traits, while also investigating their functional roles. Additionally, another Chinese study [9] aimed to develop a polygenic risk score (PRS) for evaluating its association with the occurrence of OSA in the Chinese Han population. Although a limited number of previous studies have explored co-inherited risk loci for OSA, the precise identification of the risk genes associated with OSA remains predominantly elusive. Recently, several successful research studies [10,11] have employed transcriptome-neuroimaging spatial correlation analyses to investigate neuropsychiatric disorders. These studies have yielded novel insights into the association between regional variations in brain gene expression and neuroimaging characteristics. Consequently, it is plausible to consider neuroimaging features of OSA as intermediate phenotypic modalities with genetic underpinnings. However, no prior investigations utilizing transcriptomic imaging techniques in OSA have been conducted to explore gene expression profiles and brain function-associated structures.

Our investigation utilizes resting-state MRI and 3D T1-weighted magnetization-prepared rapid gradient-echo (MPRAGE) sequence MRI data to examine the structural and functional alterations in the brains of individuals with OSA. The present study employs the resting-state functional metric ALFF and the structural metric gray matter volume (GMV) to investigate these alterations, which may be key nodes in the brain’s cognitive function and emotional regulation networks. In addition, we explored correlations between neuroimaging metrics and neurocognitive assessment tools as well as sleep parameters. Furthermore, we ascertain the correlated gene expression profiles with alterations in brain structure and function in patients with OSA through spatial correlation analysis. The gene expression data were obtained from six normal adult brain tissue samples provided by the Allen Human Brain Atlas (AHBA). Therefore, our study further links the changes in brain function and structure of OSA patients with gene expression profiles and neurocognitive function. As such, it may offer novel insights into the intrinsic molecular genetic mechanisms underlying alterations in brain structure and function among individuals with OSA, as well as shed light on the mechanism behind the heightened risk of cognitive impairments and psychiatric disorders observed in this population.

## 2. Materials and Methods

### 2.1. Participants

**Figure 1:**
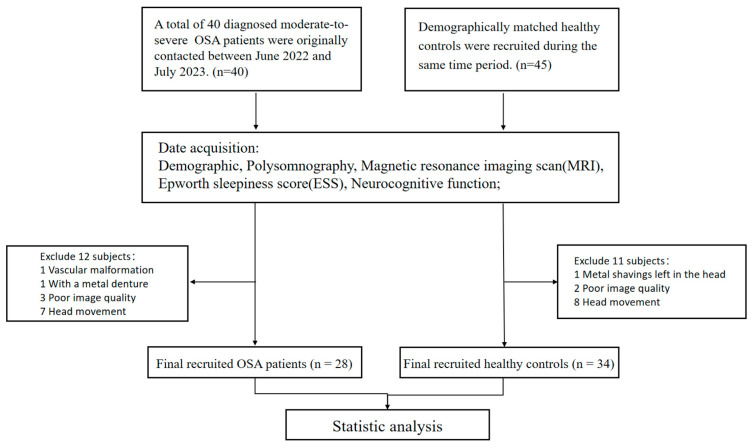
The participant recruitment process flowchart. Abbreviations: OSA, obstructive sleep apnoea.

Between June 2022 and July 2023, a total of 40 newly diagnosed patients with OSA were initially recruited from the Sleep Disorders Center at the Second Xiangya Hospital, Central South University of China. Additionally, 45 age-, gender-, and education-matched healthy controls were enrolled from the Health Management Center at the same hospital. Eventually, following essential adjustments, 12 subjects were excluded from the OSA group, including one patient with vascular malformations, one with metal dentures, three with poor image quality, and seven with severe head movements. Eleven subjects were excluded from the HC control group, excluding one patient with residual metal fragments in the brain, two patients with poor image quality, and eight patients with head movements. Finally, a total of 28 patients with OSA and 34 healthy controls were successfully enrolled who were matched in terms of age, gender, and education (Figure 1).

The inclusion criteria for patients with OSA necessitated an apnea-hypopnea index (AHI) exceeding 15 events per hour. The exclusion criteria for all participants were as follows: (1) presence of structural brain abnormalities, such as brain tumors, cerebrovascular disease, or brain infections, except for cerebral white matter high signal on conventional neuroimaging; (2) severe physical ailments including poorly managed diabetes mellitus, high blood pressure, elevated cholesterol levels, and excessive uric acid levels along with their associated severe complications; (3) significant psychiatric or neurological conditions like major depressive disorder; (4) history of traumatic brain injury and cranial surgery or substance abuse; (5) previous evaluation using comparable or indistinguishable cognitive measures; (6) failure to complete MRI scans and questionnaires; (7) head movement exceeding 2 mm in translation or 2° in rotation, or poor image quality.

All individuals in the control group, with the exception of potential obesity, exhibited normal results on physical examinations and laboratory tests within the typical range. Healthy controls were excluded if they received a polysomnography diagnosis indicating any form of sleep-disordered breathing. Additionally, all participants were right-handed native Chinese speakers aged 18 years or above. The study was conducted with the approval of the Ethics Committee of the Second Xiangya Hospital, Central South University. All procedures were performed in accordance with the guidelines outlined in the Declaration of Helsinki. Prior informed consent was obtained from all individuals involved, who also provided written consent forms.

### 2.2. Polysomnography (PSG) and Questionnaires

Diagnostic polysomnography (PSG) was conducted on all participants (Embla S4000; Medcare Technologies, Fuquay Varina, NC, USA). The comprehensive array of tools employed in this study included a tracheal microphone for snoring recording, a pulse oximeter for monitoring oxygen saturation levels, devices for electroencephalography (EEG), electrooculography (EOG), electromyography (EMG), and electrocardiography (ECG) recordings. Additionally, a nasal pressure transducer was utilized to measure nasal airflow, while thoracic and abdominal impedance belts were used to assess respiratory effort. Furthermore, sensors were employed to evaluate leg movements and sleep positions. The evaluations are conducted exclusively by highly experienced physicians specializing in sleep disorders at dedicated sleep research facilities. According to the 2017 Clinical Practice Guidelines for adult obstructive sleep apnea [12] issued by the American Society of Sleep Medicine, the diagnosis of OSA is collaboratively made by proficient respiratory physicians.

The participants’ neurocognitive functioning was evaluated upon admission using three standardized assessments: the Stroop Color Word Test (SCWT), Matrix Consensus Cognitive Battery (MCCB), and Montreal Cognitive Assessment (MoCA). Daily drowsiness was measured using the Epworth Drowsiness Scale (ESS). If the MoCA score dropped below 26, it was inferred that there had been a deterioration in cognitive function. The MCCB and SCWT scores were categorized into seven domains, encompassing the following cognitive abilities: information processing speed, logical reasoning, cognitive flexibility, memory retention and manipulation, problem-solving, focus/alertness, and analytical short-term verbal learning.

### 2.3. Image Data Acquisition

A 32-channel head coil was utilized in conjunction with a Siemens Skyra 3T MRI scanner (Siemens Healthcare, Erlangen, Germany) to collect all MRI data. During the scanning process, subjects were asked to try not to think about anything else while closing their eyes and not going to sleep. Furthermore, individuals assumed a supine position on their backs and wore headphones, while sponge pads were strategically positioned at the center of the head and within the coils to minimize motion artifacts resulting from head movements. The resting-state fMRI parameters were as follows: repetition time (TR)/echo time = 2000 ms/30 ms; matrix, 64 × 64; flip angle = 90°, voxel size = 3.5 mm × 3.5 mm × 3.5 mm, with a total of 37 axial slices and slice thickness set at 3.4 mm, field of view = 192 mm. Acquisition parameters for the three-dimensional T1-weighted sequence were as follows: 192 slices per slab with a 256 × 256 in-plane resolution; repetition time was set at 2.3 s, echo time at 2.32 milliseconds, slice thickness = 0.9 mm, and flip angle at 8°. The voxel size was 0.9 mm × 0.9 mm × 0.9 mm and inversion time at 900 milliseconds.

### 2.4. Resting-State fMRI Preprocessing and ALFF Analysis

The SPM software(version SPM12) and DPARSF (Resting-State fMRI processing and Analysis, (www.restfmri.net/ (accessed on 15 July 2023)) toolbox were used to preprocess resting-state MRI data. To ensure the stability of the imaging data during acquisition, we excluded the initial 10 time points and performed slice timing. We adjusted the slice times to minimize any impact from variations in acquisition times and head movement caused by whole head image data. Additionally, we normalized the data to conform to the standard MNI space using voxels measuring 3 × 3 × 3 mm^3^ in size. For smoothing purposes, a Gaussian kernel with a full width at half maximum (FWHM) of 6 mm was applied. To account for confounding factors, we conducted regression analysis on white matter signals, Friston’s 24-parameter head motion global mean signals, and cerebrospinal fluid. Furthermore, linear temporal detrending and temporal bandpass filtering within the frequency of 0.01–0.08 Hz were employed. We excluded data with head motion resulting in a displacement of more than 2 mm or a rotation of more than 2°. The ALFF values were obtained using the DPARSF toolkit. We applied a rapid Fourier transform to convert the filtered time series of each voxel into the frequency domain. The ALFF average square root was within the frequency range of 0.01–0.08 Hz for each voxel. First, we conducted a voxel-wise, one-sample *t*-test on standardized ALFF separately for individuals with OSA and HC in order to identify brain regions that exhibit significant standardized ALFF in these two groups. Next, within these identified regions, we conducted a voxel-wise two-sample *t*-test on standardized ALFF between individuals with OSA and HC while controlling for age, gender, education, and body mass index (BMI) as covariates of no interest. The sign of the t-value indicates the direction of the difference in the means, with a positive value indicating that the mean ALFF value of the difference brain region in the OSA group was greater than that of the healthy control group and a negative value indicating that the mean ALFF value of the difference brain region in the OSA group was less than that of the healthy control group. Following correction based on Gaussian random field (GRF) theory, all ALFF results adhered to a two-tailed voxel-wise *p* value < 0.001 and a cluster-level *p* value < 0.05.

### 2.5. VBM Analysis

The structural magnetic resonance imaging (sMRI) data was processed using the CAT12 software package(version CAT12.8.2), which built-in package based on spm12. The processing steps included the following: (1) correcting signal variance across voxels caused by B1 field inhomogeneity through bias correction; (2) classifying various brain tissues such as cerebrospinal fluid, white matter, and gray matter (GM) through tissue segmentation; (3) warping each individual GM tissue into Montreal Neurological Institute (MNI) space using the DARTEL algorithm for spatial normalization. This adjustment maintained the absolute GM tissue’s absolute volume (GMV), and resampling was performed to achieve a voxel size of 1.5 × 1.5 × 1.5 mm^3^; (4) smoothing the generated gray matter with an 8 mm full width at half maximum Gaussian kernel for spatial smoothing purposes. The smoothed cortical thickness images were incorporated into a general linear model at the group level, with covariates controlling for age, gender, education, and BMI. To identify specific regions of the brain with significant case–control differences in GMV, an independent 2-sample *t*-test was conducted. Significance levels were determined using 1000 Monte-Carlo simulations at voxel level *p* value < 0.001 and corrected cluster-wise *p* value < 0.05.

### 2.6. Meta-Analytical Decoding

**Figure 2:**
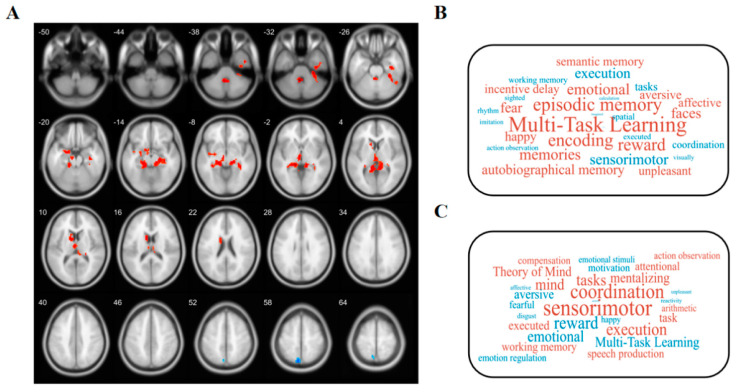
Functional decoding of ALFF-altered and GMV-changed brain areas. (**A**) Red and blue colors, respectively, represent areas of significantly higher and lower ALFF in OSA patients compared to healthy controls. After Gaussian random field (GRF) theory correction, all statistical significance of ALFF complied with a 2-tailed voxel-wise *p* value < 0.001 and a cluster-level *p* value < 0.05. The number in the upper left corner of the brain represents the z-coordinate in MNI spatial coordinates. (**B**) Word clouds showing cognitive terms associated with ALFF and (**C**) GMV brain differences using the Neurosynth Red and blue represent cognitive terms associated with regions that show significantly higher and lower ALFF/GMV, respectively.

To investigate the cognitive associations of brain regions that exhibited significant differences in ALFF and GMV between individuals with OSA and healthy controls, we performed a meta-analysis utilizing the Neurosynth database (https://neurosynth.org/decode/ (accessed on 6 August 2023)). By inputting the original spmT map, the website automatically generates terms linked to modified brain regions along with correlation coefficients, based on their values. After excluding anatomical terms, we selected the top 30 terms for presentation in a word cloud (with word size indicating correlation strength) (Figure 2B,C).

### 2.7. Brain Gene Expression Data Processing

The gene expression data were derived from the Allen Human Brain Atlas [13], which was derived from six postmortem donated adult brains. Since only two donors had whole brain tissue samples available, our analysis focused specifically on the left hemisphere. Concisely, the preprocessing of gene expression data based on the abagen [14] toolkit (https://www.github.com/netneurolab/abagen (accessed on 7 August 2023)) can be summarized [15] as follows: (1) revising probe-to-gene annotations; (2) applying an intensity-based filter; (3) selecting specific probes; (4) matching samples to brain regions; (5) managing missing data; (6) normalizing sample values; (7) normalizing gene values; (8) combining sample values for each region; and finally, (9) identifying stable genes. As a result of these preprocessing steps, we retained a total of 15,897 genes for further analysis. To segment the brain into distinct regions, we utilized a gray matter mask and generated a resulting gene expression matrix consisting of 1520 regions × 15,897 genes.

### 2.8. Gene Expression–Neuroimaging Spatial Correlation Analyses

To obtain the genes most associated with ALFF and GMV changes in OSA, we utilized statistical maps of brain imaging metrics of ALFF values and GMV values between the OSA patients and healthy subjects, and extracted t-values of 1520 samples (gray masking features) in the statistical maps based on the corresponding gray matter masks. Consequently, a T-value matrix with dimensions of 1520 samples × 1 was generated. Next, we performed Pearson correlation analysis between the gene expression matrix (1520 samples × 15,897 genes, obtained in brain gene expression data processing analysis) and T-value matrix (1520 samples × 1) using MATLAB’s core function, ultimately identifying genes that exhibited similar distributions in both gene expression values and T-values. To identify significantly associated genes, we used Bonferroni-corrected Pearson correlation analysis of *p* < 0.01 and |Pearson r| > 0.2 as significant gene inclusion criteria.

### 2.9. Pathway Analysis

Gene enrichment analysis was performed on genetic crossover samples in which both ALLL and GMV changes were significantly correlated. We performed a Gene Ontology (GO) analysis for molecular function, cellular components, and biological processes via clusterProfiler (version 4.6.2), org.Hs.eg.db (version 3.16.0), enrichplot (version 1.18.4), circlize (version 0.4.15), complexHeatmap (version 2.14.0), and ggplot2 (version 3.4.4) [16] packages.

### 2.10. Statistical Analysis

The statistical analysis was conducted using SPSS software (version 26.0, IBM Corp, Armonk, NY, USA). Categorical variables were presented in terms of absolute and relative frequency. The median and interquartile range (IQR) were utilized for continuous variables. The dissimilarities among groups were assessed using appropriate statistical techniques for continuous variables (Mann–Whitney U test) and categorical data (Fisher’s exact test). Spearman correlation was applied to evaluate the correlation between imaging indices (ALFF and GMV) and clinical parameters (including demographics, sleep parameters, and neurocognitive parameters). The results of the correlation analysis were corrected using the False Discovery Rate (FDR) method, setting the significance level at *p* < 0.05.

## 3. Results

### 3.1. Demographic and Clinical Characteristics

Significant differences were observed in body mass index (BMI), ESS scores, AHI, mean percutaneous oxygen saturation (MSpO2), N3 period, lowest percutaneous oxygen saturation (LSpO2), apnea-hypopnea index during non-rapid eye movement stage (NREM-AHI), oxygen desaturation index (ODI), apnea-hypopnea index during rapid eye movement stage (REM-AHI), sleep efficiency, and arousal index between the OSA group and HCs group. These differences were statistically significant with *p* < 0.05. Conversely, there were no statistically significant differences observed in age, REM period, gender distribution, educational background, smoking status, Trial Making Test-A, and Symbol Coding between the groups (all *p* > 0.05). As presented in Table 1, the neurocognitive evaluations conducted on individuals with OSA indicated poorer performance (*p* < 0.05) across various measures such as MoCA scale score, Stroop word test, Stroop color test, Hopkins Verbal Learning Test—Revised (HVLT-R), Stroop color-word test (SCWT), mazes, category fluency, Continuous Performance Test-identical Pairs (CPT-IP), Brief Visuospatial Memory Test-Revised (BVMT-R), and spatial span (WMS-IIISS).

### 3.2. Altered ALFF

Compared with the HCs, the OSA group showed increased ALFF values in the left hippocampus (t = 5.294, *p* < 0.05), left amygdala (t = 4.176, *p* < 0.05), left caudate (t = 4.659, *p* < 0.05), left Cerebullm (t = 5.896, *p* < 0.05), and decreased ALFF Values at left precuneus (t = −4.776, *p* < 0.05) (Table 2 and Figure 2A). In addition, regions exhibiting an increased ALFF in the OSA group were associated with muti-task learning, memory, encoding, reward, and emotional, while those exhibiting a decreased ALFF were involved in sensorimotor, execution, tasks, coordination, spatial, and working memory (Figure 2B).

### 3.3. Changed GMV

Compared with the HCs, the OSA group showed increased GMV values in the right Inferior parietal (but supramarginal and angular gyri) (t = 5.158, *p* < 0.05) (Table 2). The regions of increased GMV in the OSA group were mainly associated with sensorimotor, coordination, execution, tasks, mind, and working memory (Figure 2C).

### 3.4. Transcription–Neuroimaging Associations

Transcriptome–neuroimaging spatial correlation analyses demonstrated that the 479 gene levels of expression (top five positively correlated genes: MTMR2, EPN3, EIF5A2, RCAN2, RAMP3; top five negatively correlated genes: RIIAD1, PLPPR4, LYRM9, PGM2L1, GABRA5) were significantly associated with GMV changes, and 1547 gene expression measures were significantly related to ALFF alterations in OSA (top five positively correlated genes: PLPPR4, FAM171B, UCHL3, TMEM158, APOC1; top five negatively correlated genes: LOC100506388, NFIC, DOK3, SYT2, ADAM23).

### 3.5. Pathway Analysis

**Figure 3:**
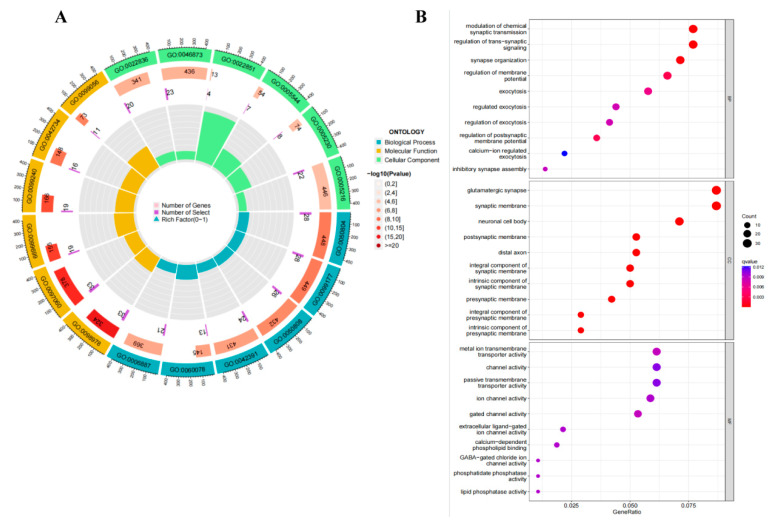
GO analysis for genetic crossover samples associated with both ALFF and GMV changes. GO analysis for genes associated with both ALFF and GMV changes in OSA patients compared to healthy controls showed significant functional enrichment in molecular functions, biological processes, and cellular components. (**A**) The results of the GO enrichment analysis are shown in Circos plots. (**B**) GO enrichment analysis results are shown as bubble plots. Abbreviations: MF, molecular functions; CC, cellular component; BP, biological process; colors indicate q-value.

To characterize the functional properties of genes associated with ALFF and GMV changes in the OSA group, we enriched their gene expression profile. The 427 genes in genetic crossover samples associated with both ALFF and GMV changes in OSA showed significant functional enrichment in molecular functions as follows: gated channel activity, metal ion transmembrane transporter activity, GABA-gated chloride ion channel activity, and calcium-dependent phospholipid binding. In the biological process, it was as follows: modulation of chemical synaptic transmission, regulation of trans-synaptic signaling, synapse organization, regulation of membrane potential and postsynaptic membrane potential. In cellular components, it was as follows: glutamatergic synapse, synaptic membrane, neuronal cell body, neuron spine, and exocytic vesicle (Figure 3A,B).

### 3.6. Correlation Analyses

**Figure 4:**
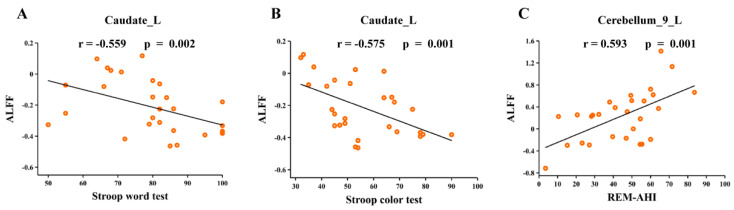
Correlation between neuroimaging metric alterations and clinical parameters. (**A**) ALFF values in the left caudate were significantly negatively correlated with the Stroop word test in OSA; (**B**) ALFF values in the left Stroop color test were significantly negatively correlated with the Stroop color test in OSA; (**C**) ALFF values in the left cerebellum were significantly positively correlated with REM-AHI in OSA. Abbreviations: ALFF, amplitude of low-frequency fluctuation; REM-AHI, apnea-hypopnea index during rapid eye movement stage; L, left; OSA, obstructive sleep apnoea.

Correlation analyses revealed that ALFF values in the left caudate (Caudate_L) were significantly negatively correlated with the Stroop word test (r = −0.559, *p* = 0.002) and Stroop color test (r = −0.575, *p* = 0.001) in OSA (Figure 4A,B). The ALFF values in the left cerebellum (Cerebellum_9_L) were positively correlated with the apnea-hypopnea index during the rapid eye movement stage (REM-AHI) (r = 0.593, *p* = 0.001) in OSA (Figure 4C).

## 4. Discussion

Obstructive sleep apnea (OSA) has been extensively documented to elicit alterations in both the structure and function of the brain [7]. By utilizing multimodal integrated structural and functional brain imaging analysis in conjunction with Allen transcriptional data from the Human Brain Atlas, we conducted an investigation into alterations in ALFF and GMV as well as their corresponding gene transcriptional profiles among moderate-to-severe OSA patients. The analysis of ALFF and VBM demonstrated changes in brain regions related to cognitive function, emotional state, and sleep regulation, which is consistent with previous research indicating that sleep disorders have a detrimental impact on cognitive function and mood regulation [17].

The common causes of sleep disorders include traumatic brain injury (TBI), psychosomatic factors, as well as medications and environmental factors. Moreover, sleep disorders increase the prevalence of hypertension, obesity, cardiovascular disease, and stroke [18]. These findings prompt our focus on elucidating the underlying mechanisms connecting OSA with these conditions. In our study, genes associated with changes in ALFF and GMV in OSA were identified by the spatial correlation between transcription and neuroimaging. Further functional enrichment analyses revealed that genes co-associated with ALFF and GMV cross-sampling exhibited enrichment in gated channel activity and synaptic transmission, as well as glutamatergic synapses and neurons. The aforementioned findings offer a fresh perspective for investigating the association between sleep characteristics and neuropsychiatric disorders, as well as unraveling the biological foundations of brain morphology and functional phenotypes in individuals with OSA.

### 4.1. Brain Regions with Altered ALFF Values

Compared to healthy controls, patients with OSA exhibited significant alterations in ALFF across various brain regions. Specifically, these changes manifested as increased ALFF values in the left hippocampus, left amygdala, left caudate nucleus, and left cerebellum, along with decreased ALFF values in the left precuneus. Patients with OSA often experience recurrent apnea and oxygen deprivation [19], which may lead to abnormal changes in ALFF values in the brain regions as an adaptive response to inadequate oxygen supply [20]. Such changes may result in decreased cognitive regulation and an increased risk of emotional problems. The precuneus [21] plays a crucial role in the integration and processing of information, encompassing visuospatial imagery, extraction of contextual memories, self-referential thinking, and awareness. A study has revealed the presence of amyloid beta accumulation in both the precuneus and posterior cingulate regions among individuals diagnosed with OSA, potentially exerting a significant influence [22] on subsequent cognitive decline. The hippocampus is susceptible to damage induced by intermittent hypoxia [23] and plays a pivotal role in the acquisition, consolidation, and retrieval of novel memories [24]. Previous studies have extensively investigated the impact of OSA on the hippocampus. For instance, one study [25] examined hippocampal volume in overweight and obese adolescents with OSA and found it to be larger compared to a control group. Furthermore, another study [26] observed disrupted functional connectivity between the hippocampus, caudate nucleus, and OSA along with elevated levels of anxiety and depressive symptoms when compared to healthy individuals. The left amygdala plays a crucial role [27] in regulating emotions. Thus, the decreased ALFF in OSA patients may potentially induce symptoms of anxiety and depression. A study has indicated that male individuals with severe OSA exhibit an atypical functional connectivity pattern within a specific subregion of the amygdala [28]. Moreover, this aberrant pattern may contribute to the presence of emotional and cognitive impairments observed in males with severe OSA, thereby further supporting our findings. The hippocampus and amygdala are both components of the limbic system, which comprises interconnected cortical and subcortical structures. This intricate system serves to integrate emotions, cognition, and behavior while also playing a crucial role in memory consolidation during sleep [29,30]. Impairment to the limbic system [31] often leads to mental disorders such as hallucinations as well as disturbances in mood and memory. Thus, sleep disorders can increase the risk of psychiatric disorders [32], due to the fact that sleep is crucial for brain stabilization and emotional regulation. The effects of sleep disorders are not limited to their direct impact on brain region activity but extend to broader areas related to mental health.

The results of correlation analysis showed that there was a significant negative correlation between ALFF values in the caudate nucleus and the Stroop Color and Word Test (SCWT) scores in patients with OSA. Meanwhile, there was a significant positive correlation between ALFF values in the cerebellum and REM-AHI. These results provide important insights into the effects of OSA on brain function and the associations with cognitive and sleep parameters.

The caudate region plays an important role in many cognitive functions [33]. The color-word test [34] is commonly used to assess the ability to inhibit cognitive interference. Damage to the caudate nucleus may affect the cognitive control and inhibition associated with the SCWT scores, leading to prolonged reaction times and increased error rates in OSA patients. The positive correlation between cerebellar ALFF values and REM-AHI may reflect a strong link between sleep parameters and brain function. The cerebellum, which plays an increasingly crucial role in regulating sleep-wake cycles, has been discovered to be the network responsible for controlling sleep and wakefulness. Additionally, it is actively involved in activities that vary depending on the stage of sleep [35]. The cerebellum is susceptible to hypoxia or ischemia, and sleep deprivation may interfere with cerebellar function [36]. The phase of sleep known as rapid eye movement (REM) [37] is a slumber typically linked to the occurrence of dreams and emotional processing. Thus, a higher REM-AHI may involve a decrease in the availability of oxygen to the patient during the REM sleep period, which may result in the functional abnormality of the cerebellum.

### 4.2. Brain Regions with Altered GMV Values

The increased gray matter volume in the inferior parietal lobule (IPL) observed in patients with OSA may exhibit a multifaceted relationship with neurophysiological mechanisms, cognitive functioning, and other cerebral alterations. The incongruity between this condition and the altered ALFF values in specific brain regions could potentially reflect the intricate nature of brain dynamics.

The inferior parietal lobule (IPL) [38] plays a crucial role in various cognitive functions such as spatial perception, sensory perception, integration of multiple senses, and mathematical processing. In turn, cognitive control [39] (or executive control) is believed to be particularly susceptible to sleep. Patients with OSA typically experience multiple episodes of apnea and inadequate oxygen supply, which negatively affects the brain. Increased gray matter volume may be a compensatory mechanism [40] in an attempt to cope with multiple hypoxic events. The brain is a highly complex organ, and different regions may respond to physiological and environmental changes in different ways [41]. Therefore, the increase in gray matter volume and the change in ALFF values may not be consistent, as they may reflect different strategies and adaptive responses of the brain in the face of factors such as OSA. In addition, brain plasticity and compensatory mechanisms may differ between regions.

### 4.3. Gene Enrichment Analysis

The spatial expression profiles of 427 ALFF and GMV co-associated cross-sample genes were closely correlated with changes in ALFF values as well as GMV values, suggesting that ALFF and GMV are involved in complex multigene interactions in OSA. These cross-sample gene enrichment results were highly enriched in gated channel activity and synaptic transmission as well as glutamatergic synapse and neuron. This implies that changes in the expression profiles of these genes may be one of the potential causes of structural and functional abnormalities in the brain. Furthermore, emerging evidence implicates sleep in the most basic of neurological functions, namely the exchange of metabolic wastes associated with neurological homeostasis [17].

According to the previous study [42], ion channels have been found to be crucial in regulating motor activity and sleep-wake cycles on a circadian basis. The modulation of ion channel characteristics by circadian rhythms influences the activation of neurons and the transmission synapses, thereby collectively governing alterations in circadian rhythm. OSA-gated channel activity may be disturbed, which may affect neuronal excitability and inhibition [43]. This may lead to a decline in cognitive function, such as difficulties with learning and memory. GABA-gated chloride channel activity in GABA [44] is an inhibitory neurotransmitter that inhibits neuronal excitability by regulating the activity of chloride channels. In OSA, abnormal activity of these channels may lead to difficulties in emotion regulation [45]. Glutamate is involved in initiating and maintaining the sleep/wake cycle while also playing a significant role in regulating rapid eye movement sleep [46]. Glutamate [47] is an excitatory neurotransmitter that may play a role in cognitive and emotional regulation. In OSA, glutamatergic synapses may be abnormally regulated. According to one study, the association between poor sleep quality and increased severity of positive symptoms, as well as decreased levels of anterio cingulate glutamate, has been reported in patients with schizophrenia [48]. In addition, synapses and neurons are critical for signaling between neurons and maintaining cognitive function [49,50]. Insufficient sleep causes modifications in molecular signaling and gene expression and potentially results in alterations to the synaptic structure of neurons [51]. Patients with OSA may have abnormal regulation of synaptic transmission, which may lead to signaling problems between neurons, affecting cognitive and emotional regulation [52].

Our study suggests that complex polygenic genetic mechanisms contribute to brain morphologic and functional abnormalities in OSA. The present study integrates changes in brain imaging and gene expression profiling to provide a comprehensive understanding of the underlying mechanisms behind brain abnormalities in OSA patients. These findings establish a crucial foundation for future research and clinical practice (particularly neuroprotective agent therapy [18]), with the potential to enhance diagnostic and therapeutic approaches for individuals with OSA, offering novel insights into the relationship between genes and brain function in this population.

### 4.4. Limitations

There are several limitations worth noting in this study. Firstly, this study is a cross-sectional observational study with a small sample size; the conclusions we reached can only be interpreted with a degree of caution, so future studies with larger sample sizes as well as longitudinal studies are needed to further validate our results. Secondly, a higher BMI was observed in patients with OSA compared to healthy controls, suggesting that obesity may influence resting-state brain activity. Although we controlled for obesity as a covariate in our brain imaging analysis, the role of obesity in MRI imaging changes in OSA needs to be further investigated. Thirdly, in transcriptome neuroimaging spatial correlation analysis, gene expression data and neuroimaging data come from different subjects, and some genes are sure to be missed due to individual differences. Finally, because the anxiety and depression scales were not used in this experiment, it was not possible to further explore the relationship between depression and anxiety conditions and cognitive and neuropsychiatric disorders in OSA subjects.

## 5. Conclusions

The present study revealed morphological and functional changes in brain regions associated with cognitive function, mood regulation, and sleep control in moderate-to-severe OSA patients. Additionally, we identified spatial correlations between gene expression profiles and these changes. These findings offer novel insights into the intrinsic molecular genetic mechanisms underlying alterations in brain structure and function among individuals with OSA, as well as shed light on the mechanism behind the heightened risk of cognitive impairments and psychiatric disorders observed in this population.

## Figures and Tables

**Table 1:** Comparison of characteristics in demography, sleep parameters, and neurocognitive tests between OSA patients and HCs.

Demographic	OSA (n = 28)	HCs (n = 34)	*p* Value
Age (year)	48 (36–53)	41 (33–51)	0.731
Gender (male) (%)	25 (89.3)	26 (76.5)	0.317
BMI (kg/m^2^)	27.9 (26.7–30.5)	24.2 (22.3–26.4)	<0.001
Education (year)	15 (12–16)	15 (12–16)	0.344
Smoker (%)	19 (67.9)	17 (50.0)	0.200
ESS scores	10.0 (7.0–16.0)	3.0 (2.0–4.5)	<0.001
Sleep efficiency (%)	84.6 (75.5–89.8)	89.5 (86.2–91.3)	0.004
N3 period (%)	6.7 (1.2–13.3)	18.6 (12.4–20.4)	<0.001
REM period (%)	18.4 (14.8–21.8)	20.3 (17.0–24.9)	0.157
AHI (events/hour)	57.0 (31.8–76.4)	3.9 (3.3–4.6)	<0.001
REM-AHI (events/hour)	47.2 (28.4–59.1)	2.7 (2.0–3.5)	<0.001
NREM-AHI (events/hour)	53.6 (29.9–71.8)	3.8 (3.1–4.7)	<0.001
LSpO2 (%)	75.5 (58.8–85)	92.0 (91.5–95.2)	<0.001
MSpO2 (%)	94.0 (92.0–96.0)	96.0 (95.5–97.0)	<0.001
ODI (events/hour)	45.4 (24.1–70.6)	3.1 (1.8–4.5)	<0.001
Arousal Index (events/hour)	45.3 (27.7–71.3)	10.8 (6.7–15.5)	<0.001
MoCA	22.5 (20.0–26.0)	25.0 (24.0–27.0)	0.003
Trial Making Test-A	37.0 (29.3–47.8)	34.0 (29.0–41.5)	0.442
Symbol Coding	39.0 (27.0–47.8)	38.0 (36.0–43.5)	0.723
HVLT-R	16.0 (14.3–22.0)	21.0 (18.5–24.5)	0.004
Spatial Span	16.0 (15.0–19.0)	19.0 (17.5–22.0)	0.002
Mazes	12.5 (10.0–18.0)	19.0 (17.5–22.0)	<0.001
BVMT-R	16.0 (12.0–22.0)	24.0 (21.0–28.0)	0.002
Category Fluency	17.0 (15.0–21.5)	22.0 (20.0–26.0)	0.001
CPT-IP	2.2 (1.4–2.6)	2.7 (2.3–3.1)	0.031
Stroop word test	81.0 (69.5–88.5)	92.0 (86.0–100.0)	0.001
Stroop color test	53.0 (45.0–68.8)	75.0 (69.0–80.0)	<0.001
SCWT	32.5 (25.5–37.8)	39.0 (32.5–44.0)	0.005

For comparison, Fisher’s exact test was used for categorical variables, and the Mann–Whitney U test was employed for continuous variables; the italic *p*-values indicate statistical significance. OSA, obstructive sleep apnea; HCs, healthy controls; BMI, body mass index; ESS, Epworth sleepiness score; AHI, apnea-hypopnea index; REM-AHI, apnea-hypopnea index during rapid eye movement stage; NREM-AHI, apnea-hypopnea index during non-rapid eye movement stage; LSpO2, lowest oxygen saturation; MSpO2, mean oxygen saturation; ODI, oxygen desaturation index; MoCA, Montreal Cognitive Assessment; HVLT-R, Hopkins Verbal Learning Test-Revised; BVMT-R, Brief Visuospatial Memory Test-Revised; CPT-IP, Continuous Performance Test-identical Pairs; SCWT, Stroop color-word test.

**Table 2:** Brain regions with abnormal ALFF and GMV between the groups.

Indices	Brain Regions	L/R	Peak MNI Coordinates			Cluster Size	t-Value
			X	Y	Z		
ALFF	Hippocampus	L	−15	−33	−3	41	5.294
	Amygdala	L	−21	−3	−18	25	4.176
	Caudate	L	−12	6	12	67	4.659
	Precuneus	L	−6	−72	57	50	−4.776
	Cerebellum_9	L	−3	−48	−33	25	5.896
GMV	Parietal_Inf	R	44	−59	60	106	5.158

The sign of the t-value indicates the direction of the difference in means, with a positive value indicating that the mean ALFF or GMV value of the brain region in the OSA group was greater than that of the healthy control group and a negative value indicating that it was less. Abbreviations: GMV, gray matter volume; ALFF, amplitude of low-frequency fluctuation; L, left; R, right; Parietal_Inf = Inferior parietal (but supramarginal and angular gyri).

## Data Availability

Data supporting the results of this study are available from the corresponding author, Jun Liu, upon reasonable request.
